# *Trichomonas vaginalis* infection and risk of cervical neoplasia: A systematic review and meta-analysis

**DOI:** 10.1371/journal.pone.0288443

**Published:** 2023-07-12

**Authors:** Andarz Fazlollahpour-Naghibi, Kimia Bagheri, Mustafa Almukhtar, Seyed Reza Taha, Mahdieh Shariat Zadeh, Kimia Behzad Moghadam, Mehrdad Jafari Tadi, Safoura Rouholamin, Maryam Razavi, Mahdi Sepidarkish, Ali Rostami

**Affiliations:** 1 Infectious Diseases and Tropical Medicine Research Center, Health Research Institute, Babol University of Medical Sciences, Babol, Iran; 2 Harlem Medical Center, Bridgeview, IL, United States of America; 3 Oncopathology Research Center, Iran University of Medical Sciences, Tehran, Iran; 4 Independent Researcher, Former University of California, San Francisco (UCSF), San Francisco, CA, United States of America; 5 School of Medicine, Shahid Beheshti University of Medical Sciences, Tehran, Iran; 6 Department of Obstetrics and Gynecology, School of Medicine, Isfahan University of Medical Sciences, Isfahan, Iran; 7 Department of Obstetrics and Gynecology, School of Medicine, Zahedan University of Medical Sciences, Zahedan, Iran; 8 Department of Biostatistics and Epidemiology, School of Public Health, Babol University of Medical Sciences, Babol, Iran; Sapienza University of Rome: Universita degli Studi di Roma La Sapienza, ITALY

## Abstract

**Objectives:**

The evidence in the literature regarding the relationship between *Trichomonas vaginalis* (*TV*) infection and cervical neoplasia is conflicting. The main aim of this study was to evaluate the magnitude of the risk of cervical neoplasia associated with *TV* infection.

**Methods:**

A meta-analysis of observational studies, which provided raw data on the association of *TV* infection with cervical neoplasia, was performed. For this aim, we searched scientific databases (PubMed/Medline, Scopus, the Web of Sciences, and Embase) from inception to March 15, 2023. A random-effects model was applied by Stata 17.0 to calculate the pooled and adjusted odds ratios (ORs) with 95% confidence intervals (CI), including subgroup, sensitivity, and cumulative analyses to explore sources of heterogeneity.

**Results:**

Of the 2584 records initially identified, 35 eligible studies contributed data for 67,856 women with cervical neoplasia, and 933,697 healthy controls from 14 countries were included. The pooled (2.15; 1.61–2.87; *I*^2^ = 87.7%) and adjusted (2.17; 1.82–2.60; *I*^2^ = 31.27%) ORs indicated a significant positive association between *TV* infection and the development of cervical neoplasia. There was no significant change in pooled and adjusted ORs by applying sensitivity and cumulative analyses, indicating the robustness of our findings. The pooled OR was significant in most sub-group analyses. There was no publication bias in the included studies.

**Conclusion:**

Our findings indicated that women with a *TV* infection are at significantly greater risk of cervical neoplasia. Future research, particularly longitudinal and experimental studies, should be done to better understand the various aspects of this association.

## Introduction

Cervical cancer is one of the leading causes of cancer deaths among women worldwide [1]. Based on global estimates in 2020, it ranks as the 4th most frequent form of cancer and the 4th major cause of cancer mortality in women [1]. More than 0.5 million cases and 0.3 million deaths have been reported in 2018 in relation to cervical cancer [2]. The most prevalent type of cervical cancer is squamous cell carcinoma (SCC) [3]. Long-term infection with the human papillomavirus (HPV) is the primary factor contributing to cervical SCC’s development [4]. Pap smear (cytology) of the cervix with HPV testing is performed in high-risk women for cervical cancer (like SCC) screening and diagnosis [5]. In the early stages of SCC, precancerous lesions are referred to as squamous intraepithelial lesions (SIL) or cervical intraepithelial neoplasia (CIN), which is categorized according to the severity of dysplasia as either low-grade or high-grade (CIN1/LSIL and CIN2-3/HSIL, respectively) [6–8]. There may, however, be some morphological characteristics that are unclear for CIN/SIL classification based on the quality of the sample. In this situation, the “atypical squamous cells” (ASCs) category is used, which can be classified into atypical squamous cells of undetermined significance (ASC-US) or high-grade cannot be ruled out (ASC-H) [9]. While high-grade lesions need management once they get confirmed with histology, monitoring and follow-up are enough for low-grade lesions because of their minimal chance of progressing into high-grade lesions and cancer [5, 10]. For this reason, it is essential to investigate the factors that contribute to the underlying processes that are involved in the development of cervical neoplasia in order to reduce the risk of cervical cancer and improve the effectiveness of treatments for the disease.

Even though HPV is an essential and recognized factor in abnormalities and cancer of the cervix, it is not a sufficient cause. There are several risk factors associated with HPV-associated cervical cancer, such as age at first sexual contact, the number of partners, smoking, oral contraceptives, the presence of human immunodeficiency virus (HIV), Herpes simplex, and co-infection with other genital or sexually transmitted infections (STIs) [11]. It is unknown why most women with HPV infection do not develop cervical neoplasia or cancer. It may be related to a lack of knowledge on vaginal infections impact on the evolution of cervical cancer [12]. It is possible that women who are infected with HPV and also have a co-infection have a decreased likelihood of spontaneous HPV clearance and progression to cancer. Epidemiological evidence shows that *Trichomonas vaginalis* (*TV*) infections may increase the risk of cervical cancer in infected women [13–15].

As a flagellated protozoan parasite, *TV* is capable of infecting both the male and female reproductive systems [16]. Approximately 270 million cases of trichomoniasis are reported each year worldwide, which is the most common non-viral STI [17]. The most significant symptoms in women are a foamy, yellow-green, unpleasant smell of vaginal discharge with genital irritation, itching, as well as pain during sexual intercourse and urination, but most of the women are asymptomatic, which affects women’s health outcomes [18]. Vaginal, cervical, urethral, and pelvic inflammation and pregnancy complications such as ectopic pregnancy, preterm delivery, and infertility are the complications that can be caused by *TV* infection [19, 20]. Although the relationship between *TV* infection and the development of cervical cancer is still not entirely understood, it is thought that the inflammatory response is the key factor [21, 22]. Inflammation is thought to make it easier for HPV to enter the epithelium’s basal layer. Thus, viral DNA is incorporated into the host genome. Moreover, *TV* infection could overexpress viral tumorigenesis, which helps activate cancer-causing pathways [23].

There are several clinical and observational studies evaluating the association between *TV* infection and cervical neoplasia [24–29]. A significant association has been shown in a combined analysis of 24 studies, which most of them were case reports or case series [30]; moreover, a meta-analysis of 17 observational studies in 2018 demonstrated a positive association between *TV* and the development of cervical cancer [31]. In this study, we determined whether the results of the new studies added had any impact on the results of the previous meta-analysis. As a result, in order to acquire a more in-depth understanding of the potential connection between *TV* infection and cervical neoplasia, we updated the previous meta-analysis.

## Materials and methods

The Preferred Reporting Items for Systematic Reviews and Meta-analyses (PRISMA) (S1 Checklist) and the Meta-analysis of Observational Studies in Epidemiology (MOOSE) reporting guidelines were followed for this systematic review and meta-analysis [32, 33]. In this study, cervical neoplasia was defined as cervical intraepithelial neoplasia ranging from atypical squamous cells of undetermined significance (ASC-US) to invasive cancer confirmed by histopathological methods.

### Search strategy and study selection

Two researchers (A.F.N. and K.B.) did a comprehensive systematic search in international scientific databases, including PubMed/Medline, Scopus, Web of Science, and Embase, for all peer-reviewed, published studies assessing the relationship between *TV* infection and cervical neoplasia from database inception to March 15, 2023. We did not use any language, geographic, or date restrictions. For database searches, the following Medical Subject Headings (MeSH) terms and keywords were combined using Boolean operators (i.e., "OR" and/or "AND"): "*Trichomonas vaginalis*", "*Trichomonas* vaginitis", "*Trichomonas* infections", "Trichomoniasis", "*Trichomonas*", "sexually transmitted infection", "cervical cancer", "cervical carcinoma", "cervical neoplasm", "cervical abnormality", "cervical dysplasia", "cancer of the cervix", "carcinoma of the cervix", and "cervical intraepithelial neoplasia" (S1 Fig). Additionally, the first 20 pages of Google Scholar and the reference list of relevant studies, including meta-analyses, were reviewed to ensure that no publications, such as gray literature, were missed. ’Google Translate’ was used to translate articles from other languages into English. Duplicate citations were removed after exporting by EndNote Reference Manager X8.

### Inclusion and exclusion criteria

After deleting duplicates, three trained researchers (K.B., K.B.M., and M.J.T.) independently screened the titles and abstracts and also evaluated the full-text of articles for eligibility. Ambiguous articles were examined by the lead investigator (AR). All the inconsistencies were resolved through discussion. Studies were eligible for inclusion if they investigated the correlation between *TV* infection and cervical neoplasia using a cross-sectional, cohort, or case-control design; gave a clear explanation of the methods for diagnosis of *TV* infection and cervical neoplasia; and had sufficient data to calculate the odds ratio (OR) or relative risk (RR) with 95% a confidence interval (95% CI). Studies that evaluated the association following the treatment, conference abstracts, reviews, systematic reviews, editorials, case reports, case series, and papers with not available full-text were excluded. In this meta-analysis, if multiple studies reported on the same population or datasets, the most recent or comprehensive study was chosen for inclusion.

### Data extraction and quality assessment

Three independent investigators (A.F.N., S.R.T., and M.S.) performed data extraction from each relevant study. Consulting with the lead investigator (A. R.) helped resolve any disputes. The following information was extracted (if available): first author’s name, publication year, country, geographic region, type of study, diagnostic method for *TV* infection, number of women with cervical neoplasia as cases, number of women without cervical neoplasia as control group, number of infected women in case and control groups. Where available, adjusted ORs were extracted and used for meta-analysis to reduce confounding, as recommended by the Cochrane Handbook [34]. According to extractable data in included studies, we categorized cervical neoplasia into the following categories: (1) ASC-US; (2) ASC-H; (3) LSIL/CIN1; (4) HSIL/CIN2/CIN3; and (5) invasive cancer. The risk of bias for each study was independently assessed by two investigators using the Joanna Briggs Institute (JBI) Critical Appraisal Tools for cross-sectional, case-control, and cohort studies, and each study was classified as having a low, moderate, or high risk of bias, as recommended for these tools [35–37].

### Data synthesis and statistical analysis

Meta-analysis was performed with Stata software version 17 (STATA Corp., College Station, TX, USA). For all tests, two-sided *P*-values were determined, with *P*-values less than 0.05 considered statistically significant. Summary pooled and adjusted ORs and related 95% confidence intervals (CIs) were calculated using the DerSimonian-Laird random-effects model (REM) for combining results across studies, which incorporates between- and within-study variance [38]. We used REM because heterogeneity was expected. Cochran’s Q test and *I*^2^ statistics were applied to measure between-study heterogeneity. An *I*^2^ value more than 75% was considered significant heterogeneity [39]. We also used subgroup and meta-regression analyses to determine the heterogeneity source. For this purpose, subgroup analyses were performed according to publication year (before 2005, 2006 to 2015, and 2016 to 2023), type of study (cross-sectional, case-control, and cohort studies), diagnostic method of *TV* infection (polymerase chain reaction (PCR), cytology, microscopy, and other), WHO-defined geographical region (Middle-East and Africa, Europe and North America, South America, Western Pacific, and South-East Asia), country’s income level (high, upper middle, and lower middle), and risk of bias for studies (low and moderate). Sensitivity analyses were performed by excluding individual studies to examine the robustness of the results and the effect of each study on the estimated ORs. Cumulative meta-analyses were applied to determine the reliability of the estimated ORs. Publication bias was evaluated using the Egger test and visual inspection of funnel plots [40].

## Results

### Study selection and characteristics

**Fig 1:**
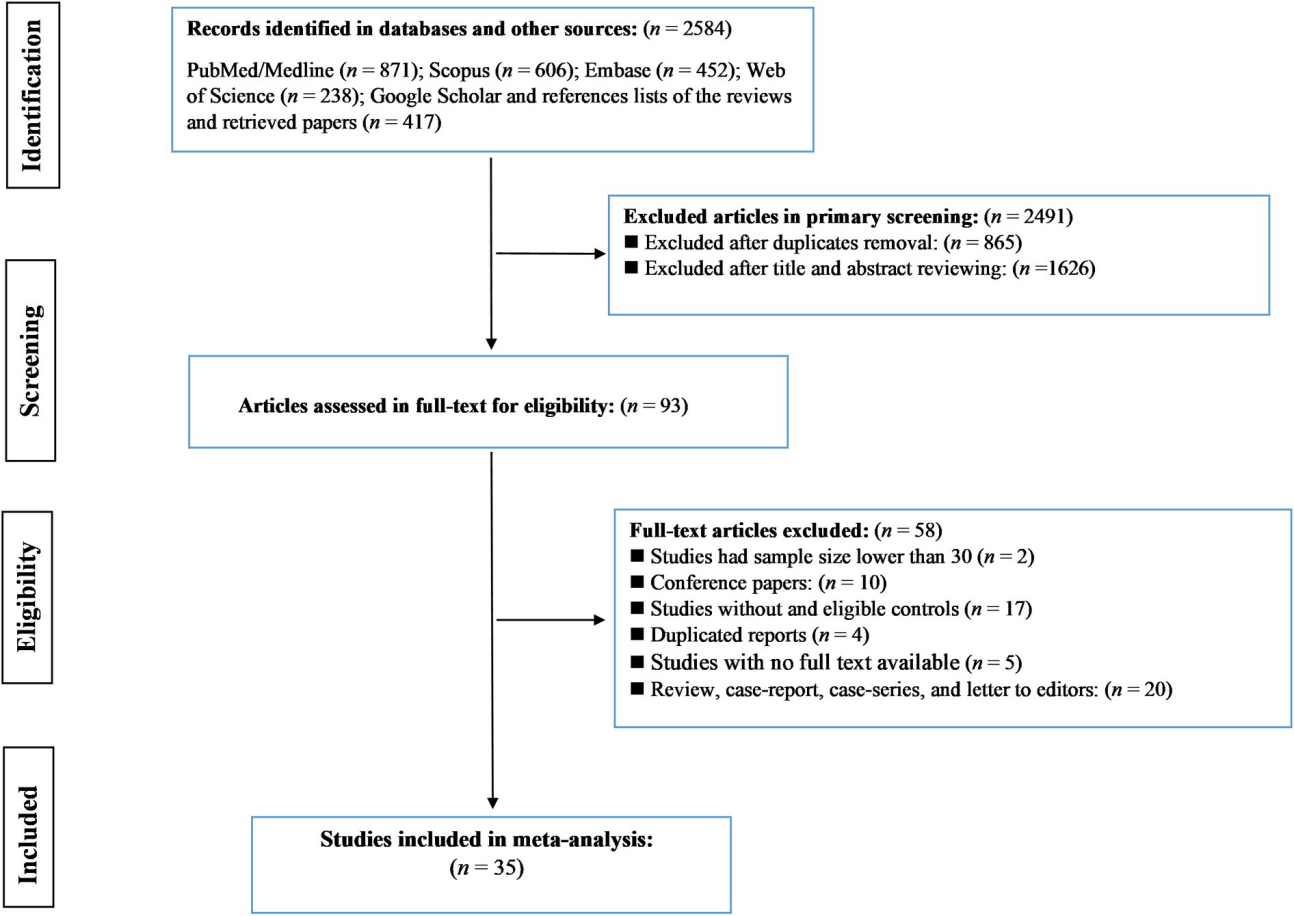
PRISMA flow chart showing study selection process.

As shown in the PRISMA flow diagram (Fig 1), our initial search in databases, Google Scholar, and bibliographies of relevant studies identified a total of 2584 records. Following the removal of 865 duplicates, an extra 1626 records were also excluded after reviewing their titles and abstracts. Ninety-three studies were eligible for full-text review. Of these, 58 articles were excluded due to missing crucial data or failing to meet the study’s inclusion criteria, thus leaving a total of 35 eligible articles [22–29, 41–67]. The studies included in the meta-analysis, published from 1985 to 2021, comprised 67,856 women with cervical neoplasia in different stages and 933,697 healthy controls. Of the 35 studies, 11 were conducted in the Western Pacific [24, 28, 29, 47, 48, 57, 59, 62–65], 9 in Europe and North America [27, 41–44, 46, 49, 51, 52], 7 in South America [23, 26, 50, 54, 55, 66, 67], 4 in South-East Asia [22, 56, 58, 60], and 4 in the Middle East and Africa [25, 45, 53, 61]. All of these studies were observational: 20 case-control studies [22, 25, 41, 42, 44–51, 54, 58, 61–65, 67], 10 cross-sectional studies [23, 24, 26–28, 55, 57, 59, 60, 66], and five cohort studies [29, 43, 52, 53, 56]. *TV* infection was assessed by cytology in 13 studies [27, 29, 43, 45, 47–49, 52, 56, 58, 61, 62, 67], PCR-based methods in nine studies [23, 25, 26, 41, 53–55, 59, 66], microscopy in eight studies [22, 24, 28, 42, 44, 50, 60, 63], and other methods (hanging drop and medical reports) in five studies [46, 51, 57, 64, 65]. Based on methodological quality, 12 and 23 studies were categorized as moderate and low risk of bias studies, respectively (Table 1). The main characteristics of the included studies are summarized in Table 1. Considering stages of cervical neoplasia, extractable data were available for ASC-US in seven studies, for ASC-H in four studies, for LGSIL/CIN1 in 14 studies, for HGSIL/CIN2/CIN3 in 17 studies, and for invasive cancer in nine studies.

**Table 1:** Characteristics of included studies.

Author	Country	Income level	Study design	Diagnostic method for *TV*	Quality assessment	Total women with cervical neoplasia	*TV*-infected	Normal women	*TV*-infected
Ghosh et al. (2017) [22]	India	LM	CC	Microscope	High	274	214	209	107
Schiff et al. (2000) [49]	USA	High	CC	Cytology	High	111	18	321	39
Su et al. (2020) [48]	Taiwan	High	CC	Cytology	High	54003	12	216012	18
Gupta et al. (2020) [58]	India	LM	CC	Cytology	High	100	6	119	2
Li et al. (2010) [47]	China	UM	CC	Cytology	High	374	51	6339	547
Gram et al. (1992) [43]	Norway	High	Cohort	Cytology	High	413	17	179555	3882
Spinillo et al. (2006) [52]	Italy	High	Cohort	Cytology	High	145	5	544	13
Dey et al. (2016) [56]	India	LM	Cohort	Cytology	High	747	78	7180	274
Zhang et al. (1995) [29]	China	UM	Cohort	Cytology	High	99	7	140018	3650
Vieira-Baptista et al. (2016) [27]	Portugal	High	CS	Cytology	High	256	7	366	14
Qin et al. (2014) [64]	China	UM	CC	Hanging drop	High	266	55	362	47
Zheng et al. (2020) [63]	China	UM	CC	Microscope	High	432	55	100	8
Li et al. (2020) [24]	China	UM	CS	Microscope	High	357	2	8733	30
Yang et al. (2020) [28]	China	UM	CS	Microscope	High	1738	309	300617	5374
Donders et al. (2013) [41]	Canada	High	CC	PCR	High	5567	42	57684	194
Lazenby et al. (2014) [25]	Tanzania	LM	CC	PCR	High	36	4	288	13
Amorim et al. (2017) [54]	Brazil	UM	CC	PCR	High	62	38	70	4
Alotaibi et al. (2020) [53]	Saudi Arabia	High	Cohort	PCR	High	95	0	255	1
Kim et al. (2016) [59]	Korea	High	CS	PCR	High	800	5	200	1
de Abreu et al. (2016) [55]	Brazil	UM	CS	PCR	High	224	19	614	78
Belfort et al. (2021) [23]	Brazil	UM	CS	PCR	High	48	21	514	86
La Vecchia et al. (1986) [46]	Italy	High	CC	Self-report	High	533	70	533	39
Feng et al. (2018) [57]	China	UM	CS	Self-report	High	267	21	11586	1170
Guijon et al. (1985) [42]	Canada	High	CC	Microscope	Moderate	32	1	52	1
Guijon et al. (1992) [44]	Canada	High	CC	Microscope	Moderate	106	4	79	4
Silva et al. (2013) [50]	Brazil	UM	CC	Microscope	Moderate	39	5	302	9
Muitta et al. (2019) [61]	Kenya	LM	CC	Cytology	Moderate	102	0	40	1
Kharsany et al. (1993) [45]	South Africa	UM	CC	Cytology	Moderate	28	11	69	15
Barcelos et al. (2011) [67]	Brazil	UM	CC	Cytology	Moderate	30	2	70	2
Qiu et al. (2017) [62]	China	UM	CC	Cytology	Moderate	40	2	40	4
Liu et al. (2015) [65]	China	UM	CC	Hanging drop	Moderate	50	21	50	7
Mohanty et al. (2020) [60]	India	LM	CS	Microscope	Moderate	152	50	48	12
Costa-lira et al. (2017) [26]	Brazil	UM	CS	PCR	Moderate	47	0	133	24
Slattery et al. (1989) [51]	USA	High	CC	Self-report	Moderate	263	53	405	21
Suehiro et al. (2021) [66]	Brazil	UM	CS	PCR	Moderate	20	1	190	0

**Abbreviation: ND,** not determined; **PCR,** polymerase chain reaction; **UM**, upper middle; **LM**, lower middle; **CC**, case-control; **CS**, cross-sectional.

### Synthesis of results

As shown in Table 2, the pooled analysis of 35 studies yielded a summary crude OR of 2.15 (95% CI = 1.61–2.87), indicating that *TV* infection was significantly associated with an increased risk of cervical neoplasia. A substantial heterogeneity (χ2 = 553.21; *I*^2^ = 87.7%) was observed between the studies. The sensitivity analysis indicated that the exclusion of any of the studies did not have a significant impact on the pooled OR (range: minimum 2.01, maximum 2.27; S2 Fig), revealing the high stability of our results. Cumulative analysis indicated that except for the first study in 1985, the pooled OR was significant for other studies (S3 Fig). Employing the Egger’s regression test and Funnel plot, we found there is no publication bias in the studies included (β = -0.71, 95% CI: -1.67, 0.25; p-value = 0.143; S4 Fig). Considering different stages of cervical neoplasia REM, indicated *TV* infection is significantly associated with ASC-US (OR, 2.88; 95% CI, 2.31–3.59), LGSIL/CIN1 (OR, 2.31; 95% CI, 1.26–4.23) and invasive cancer (OR, 2.38; 95% CI, 1.11–5.09).

**Table 2:** Sub-group analysis of the pooled prevalence and odds ratios for the association between *Trichomonas vaginalis* and cervical neoplasia.

Variables	Datasets (n)	Pooled prevalence of case % (95% CI)	Pooled prevalence of control% (95% CI)	Odds ratios (95% CI)	Heterogeneity (*I*^2^%)
**Publication year**					
Before 2005	8	11.09 (5.97–17.46)	3.85 (3.13–4.64)	2.17 (1.53–3.07)	49.67
2006–2015	8	11.54 (3.11–23.92)	4.94 (1.06–11.21)	2.04 (1.65–2.51)	9.61
2016–2023	19	10.27 (3.65–19.53)	5.84 (3.72–8.37)	1.94 (1.11–3.41)	93.91
**Type of study**					
Cross-sectional	10	8.36 (2.49–16.93)	6.03 (2.96–10.04)	1.57 (0.69–3.61)	94.18
Case-control	20	14.37 (8.09–2.01)	6.77 (4.62–9.26)	2.41 (1.81–3.19)	67.91
Cohort	5	4.36 (1.36–8.79)	2.55 (2.11–3.03)	2.54 (1.92–3.34)	16.01
**Diagnostic method**					
PCR	9	8.49 (2.73–16.67)	4.42 (0.67–10.88)	2.16 (0.76–6.13)	91.32
Cytology	13	6.38 (1.83–13.09)	4.13 (2.17–6.61)	1.93 (1.49–2.51)	42.35
Microscopy	8	16.63 (4.32–34.44)	7.73 (4.34–11.93)	2.71 (1.37–5.32)	89.32
Other	**5**	18.44 (11.69–26.29)	8.98 (6.57–11.71)	2.08 (1.11–3.92)	87.25
**WHO region**					
Middle East and African	4	6.45 (0.01–24.45)	5.11 (0.27–14.21)	2.09 (1.05–4.15)	0
Europe and north America	9	6.28 (1.92–12.69)	3.75 (2.12–5.78)	1.84 (1.29–2.63)	63.78
South America	7	15.87 (3.07–35.01)	6.89 (2.27–13.58)	2.81 (0.72–11.02)	91.13
Western pacific	11	8.62 (2.19–18.47)	4.14 (2.14–6.71)	2.14 (1.25–3.66)	92.01
South east Asia	4	29.07 (2.79–67.79)	16.03 (0.48–45.41)	2.89 (2.35–3.56)	0
**Income level**					
High	12	3.84 (1.61–6.91)	2.46 (1.08–4.34)	1.88 (1.38–2.57)	53.63
Lower middle	6	18.54 (1.34–47.85)	11.11 (1.72–26.47)	2.85 (2.33–3.51)	0
Upper middle	17	14.62 (9.36–20.74)	6.57 (5.04–8.27)	2.41 (1.39–4.13)	93.27
**Quality score**					
Low	23	10.76 (5.91–16.81)	5.11 (3.73–6.67)	2.22 (1.58–3.12)	91.25
Moderate	12	10.88 (3.81–20.59)	7.09 (3.26–12.08)	1.91 (1.04–3.47)	62.13
**Total**	35	10.79 (6.53–15.91)	5.48 (4.23–6.87)	2.15 (1.61–2.87)	87.77

**Abbreviations: CI,** confidence interval; **NA,** not applicable; **WHO,** World health organization.

Subgroup analysis considering type of studies indicated significant positive associations in case-control (OR, 2.41; 95% CI, 1.81–3.19; χ2 = 50.34; *I*^2^ = 67.9%) and cohort (OR, 2.54; 95% CI, 1.92–3.34; χ2 = 3.86; *I*^2^ = 16.01%) studies, while the association was not significant in cross-sectional studies (OR, 1.57; 95% CI, 0.69–3.61; χ2 = 303.67; *I*^2^ = 94.18%). With regard to geographical regions, studies from regions of the Middle East and Africa (OR, 2.09; 95% CI, 1.05–4.15; χ2 = 3.35; *I*^2^ = 0.0%), Europe and north America (OR, 1.84; 95% CI, 1.29–2.63; χ2 = 18.46; *I*^2^ = 63.78%), Western pacific (OR, 2.14; 95% CI, 2.35–3.56; χ2 = 323.39; *I*^2^ = 92.1%), South east Asia (OR, 2.89; 95% CI, 2.35–3.56; χ2 = 4.01; *I*^2^ = 0.0%) demonstrated significant association, while studies from South America showed non-significant results (OR, 2.81; 95% CI, 0.72–11.02; χ2 = 54.06; *I*^2^ = 91.13%). According to diagnostic methods for detection of *TV* infection, subgroup analysis indicated significant association in studies that used cytological (OR, 1.93; 95% CI, 1.49–2.51; χ2 = 23.19; *I*^2^ = 42.35%), microscopic (OR, 2.71; 95% CI, 1.37–5.32; χ2 = 103.08; *I*^2^ = 89.32%), and other (culture and medical records) (OR, 2.08; 95% CI, 1.11–3.92; χ2 = 29.52; *I*^2^ = 87.25%), while studies that used PCR showed a non-significant association (OR, 2.16; 95% CI, 0.76–6.13; χ2 = 52.89; *I*^2^ = 91.32%). The association was significant in all subgroup analyses based on publication year, income levels and risk of biases. More details on subgroup analyses are presented in Table 3.

**Table 3:** The relationship between *Trichomonas vaginalis* and cervical neoplasia based on stage of abnormality.

Type of cervical neoplasia	Pooled prevalence of case % (95% CI)	Pooled prevalence of control% (95% CI)	Odds ratios (95% CI)	Heterogeneity (*I*^2^%)
ASC-US	8.78 (1.46–20.11)	5.48 (4.23–6.87)	2.88 (2.31–3.59)	0
ASC-H	0.41 (0.01–7.28)	5.48 (4.23–6.87)	2.67 (0.51–14.19)	44.23
LGSIL/CIN1	11.61 (2.62–24.89)	5.48 (4.23–6.87)	2.31 (1.26–4.23)	75.38
HGSIL/CIN2/CIN3	8.33 (2.72–16.08)	5.48 (4.23–6.87)	1.54 (0.76–3.11)	82.97
Invasive Cancer	16.13 (1.98–38.76)	5.48 (4.23–6.87)	2.38 (1.11–5.09)	79.84
Total cervical **neoplasia**	10.79 (6.53–15.91)	5.48 (4.23–6.87)	2.15 (1.61–2.87)	87.77

**Fig 2:**
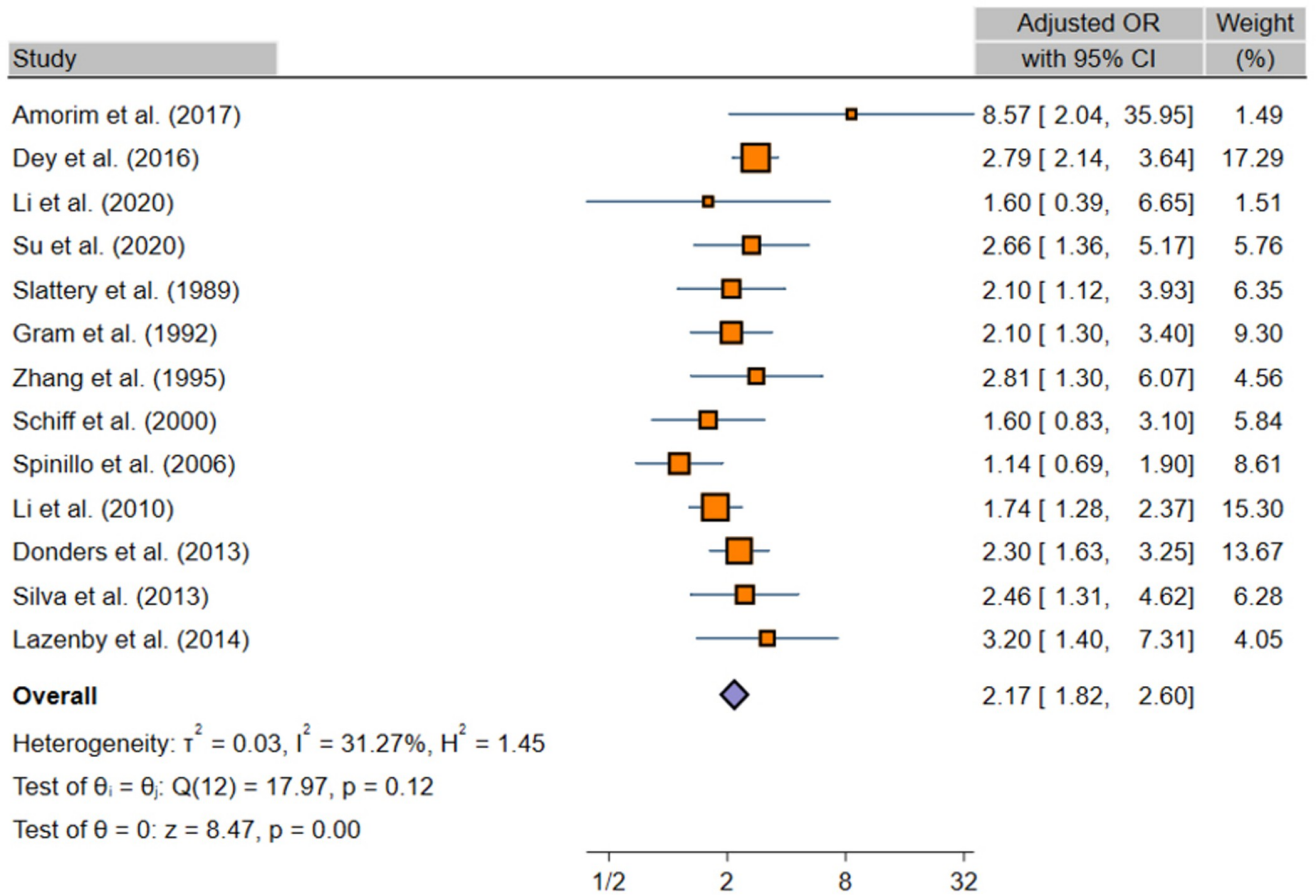
Forest plot of adjusted ORs, pooled with random effects, regarding the association between *Trichomonas vaginalis* infection and cervical neoplasia.

Thirteen studies have reported adjusted OR [24, 25, 29, 41, 43, 47–52, 54, 56], a REM on these studies also indicated significant positive association between *TV* infection and cervical neoplasia (aOR, 2.17; 95% CI, 1.82–2.60; χ2 = 17.97; *I*^2^ = 31.27%; Fig 2). Employing the Egger’s regression test and Funnel plot, we found there is no publication bias in the studies included (β = 0.67, 95% CI: -1.05, 2.40; p-value = 0.411; S5 Fig). Cumulative analysis indicated that, the pooled adjusted OR was significant throughout all the years (S6 Fig). Sensitivity analysis showed that the estimates of the pooled adjusted OR range from 2.04 (95% CI: 1.71–2.42) to 2.30 (95% CI: 1.96–2.70), suggesting that no one study is substantially influencing the pooled estimate (S7 Fig).

## Discussion

Cervical cancer incidence is rising quickly, and cervical cancer deaths are anticipated to be almost 0.5 million by 2030 globally [68]. Accordingly, this cancer is a serious health concern worldwide. Traditional treatments, such as chemotherapy, radiotherapy, and surgery, can be given for cervical cancer at an early stage, but these treatments are not efficient for late-stage cancer [69]. The recurrence rate of cervical cancer after surgery in CIN 2+ is 5–10% at 5 years [70–72]. Additionally, these treatments are unsafe. An increased risk of preterm delivery, lower birth weight, and preterm premature rupture of the membrane before 37 weeks of pregnancy have been shown to be linked with surgical treatment of the CIN [73]. As a result, despite major therapy advancements, cervical cancer mortality remains high, and the 5-year survival rate for women with advanced-stage cervical cancer is just 17% [74, 75]. These findings show the importance of finding gaps in cervical cancer risk factors to prevent this cancer, which has a high mortality rate.

In the present updated systematic review and meta-analysis of the association between *TV* infection and cervical neoplasia, we found that women with *TV* infection had a significantly higher risk of developing cervical cancer. This significant correlation was found in most subgroup analyses, including cohort and case-control studies, most geographical regions, all time periods, and studies with both moderate and low risk of bias. Consistent with our findings, a previous meta-analysis study undertaken by Yang et al. [31] demonstrated a significant potential role of *TV* in the development of cervical neoplasia and cancer. Furthermore, a combined analysis in 1994 revealed that the risk of cervical neoplasia doubled in the presence of this parasite infection [30].

Infection with *TV* could also be related to the development of other types of urogenital abnormalities, including prostate and bladder cancers [76, 77]. A previous meta-analysis investigated the correlation between prostate cancer and *TV* infection as well and found that patients with *TV* infection had a 1.17-fold higher risk of prostate cancer, indicating the potential role of *TV* infection in the development of different types of cancer [77]. Our results are consistent with those of other meta-analyses, which also found that other STIs play a significant role in cervical cancer development. For example, *Chlamydia trachomatis* infection was significantly correlated with cervical cancer in meta-analyses [78–80]. In another study, genital *mycoplasma* infection was reported to be linked to HPV-related cervical neoplasia. They demonstrated that *Mycoplasma genitalium* and *Ureaplasma urealyticum* were correlated with an increased risk of infection with high-risk HPV viruses. A significantly elevated risk of abnormal cervical cytopathology has also been linked to *Ureaplasma urealyticum*, *Ureaplasma parvum*, and *Mycoplasma hominis* [81]. It has also been shown that HIV infection is linked to slower clearance and higher HPV infection rates. As a result of increased HPV persistence in HIV patients, higher rates of LSIL and HSIL and an increased incidence of cervical cancer were observed [82]. Even though epidemiological studies found a positive correlation between HSV-2 infection and cervical cancer risk, subgroup analysis in cohort studies did not find a significant association between HSV-2 infection and cervical cancer [83]. These findings indicate that cervical cancer progression is influenced by STIs other than HPV. The role and mechanism of these infections in cervical cancer need to be studied further.

Although the exact mechanisms by which *TV* causes cervical cancer are unknown, it seems likely that it is caused by a combination of factors that work together in a complicated way. Several studies have found molecular links between *TV* infection and cervical cancer progression. Chronic inflammation of the cervix is one possible mechanism by which *TV* contributes to cervical cancer. This leads to immune cell-attracting cytokines and chemokines, increased production of reactive oxygen species, which damage DNA, and genetic mutations that raise the risk of cancer development [19, 84–86]. *TV* infection is associated with the development of high-grade CIN due to damage and alterations to the cervical epithelium, which create a conducive environment for the organism’s proliferation. The immune response generated against the invading organism involves the recruitment of numerous leukocytes, leading to inflammation, which can promote DNA damage and mutations [11, 87]. Besides, *TV* generates cytotoxic chemicals that stimulate epithelial atypia and dysplasia, such as cell-detaching factors and N-nitrosamines. The organism thrives on tissue debris and serous exudate and produces significant tissue damage [88]. The pH level in the vagina also rises during *TV* infection, which further creates a favorable environment for the organism to proliferate [89]. In addition, *TV* can interact with HPV, a known risk factor for cervical cancer. The parasite can enhance HPV’s ability to infect host cells and promote the persistence of the virus in infected cells, which can increase the risk of cervical cancer [22, 23]. Another proposed mechanism is through nitric oxide (NO). The parasite can induce the production of NO by neutrophils in the cervix, which can cause DNA damage and promote the growth of abnormal cells. This can lead to pre-cancerous lesions and eventually cervical cancer. Moreover, HPV infection has also been shown to induce NO release, which may contribute to cervical cancer [90, 91].

This study has important strengths. By including 35 studies that examined 67,856 women with cervical neoplasia and 933,697 healthy controls and applying several subgroup, sensitivity, and cumulative analyses, this study provides the most up-to-date and comprehensive evidence considering the relationship between *TV* infection and the development of cervical neoplasia. We found no significant change for the pooled and adjusted ORs in sensitivity and cumulative analyses, which indicates the robustness of our findings. This study also has some limitations. Despite our comprehensive search, it is likely that some studies—mostly those that were published in non-indexed local journals—were overlooked. The information for potentially relevant confounding factors such as age, alcohol and tobacco use, high-risk behavior in sexual activity, and co-infection with HPV, HIV, and other STIs were not available or consistently reported in most studies; therefore, we were unable to extract and analyze these covariates. Nevertheless, we analyzed adjusted ORs reported in 13 studies, so this limitation has been addressed to a large extent. Most studies were cross-sectional or case-control, and there were only five cohort studies. Therefore, it is difficult to draw conclusions about the natural history of *TV* infection and cervical neoplasia due to the lack of longitudinal data. We found substantial heterogeneity in most of the meta-analytic estimates, although low non-significant heterogeneity was recorded for cohort studies (*I*^2^ = 16%) and adjusted ORs (*I*^2^ = 31%). Variation in diagnostic methods for the diagnosis of *TV* infection, definition and stages of cervical neoplasia, geographical region, country’s income levels, and publication year are likely to have contributed to the heterogeneity (Table 3).

## Conclusion

This updated systematic review and meta-analysis confirmed findings of the previous meta-analysis and indicated that women with *TV* infection are at a higher risk of cervical neoplasia. Future research, particularly longitudinal and experimental studies, should be done to better understand various aspects of this association. Moreover, more investigation is needed to determine how *TV* interacts with other infectious agents and environmental covariates that may predispose women to cervical neoplasia, as well as to identify amenable factors and effective interventions that can be addressed by the public health system.

## Supporting information

S1 Fig Search strategy in databases. (TIF)

S2 Fig Forest plot, sensitivity analysis of studies evaluating the association between Trichomonas vaginalis and cervical abnormalities. (TIF)

S3 Fig Forest plot, cumulative analysis of studies evaluating the association between Trichomonas vaginalis and cervical abnormalities. (TIF)

S4 Fig Funnel plot, publication bias in studies evaluating the association between Trichomonas vaginalis and cervical abnormalities. (TIF)

S5 Fig Funnel plot, publication bias in studies having adjusted ORs that evaluated the association between Trichomonas vaginalis and cervical abnormalities. (TIF)

S6 Fig Forest plot, sensitivity analysis of studies with adjusted OR evaluating the association between Trichomonas vaginalis and cervical abnormalities. (TIF)

S7 Fig Forest plot, cumulative analysis of studies with adjusted OR evaluating the association between Trichomonas vaginalis and cervical abnormalities. (TIF)

S1 Checklist PRISMA checklist 2020 for ‘*Trichomonas vaginalis* infection and risk of cervical neoplasia: A systematic review and meta-analysis paper’. (DOCX)
