# Supplementary figures and images for: Trichomonas vaginalis infection and risk of cervical neoplasia: A systematic review and meta-analysis
Source: PLoS One. 2023 Jul 12;18(7):e0288443. doi: 10.1371/journal.pone.0288443 (PMC10337954; doi:10.1371/journal.pone.0288443)

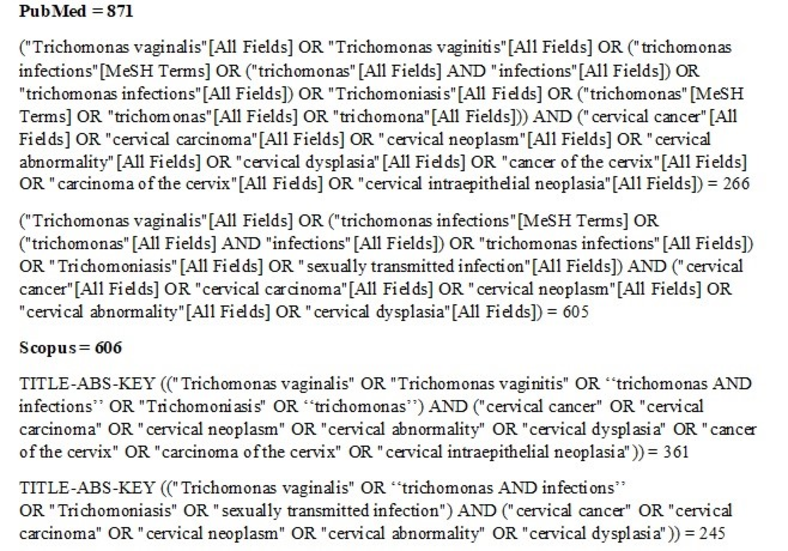

Supplement: S1 Fig — (TIF) [file pone.0288443.s001.tif]

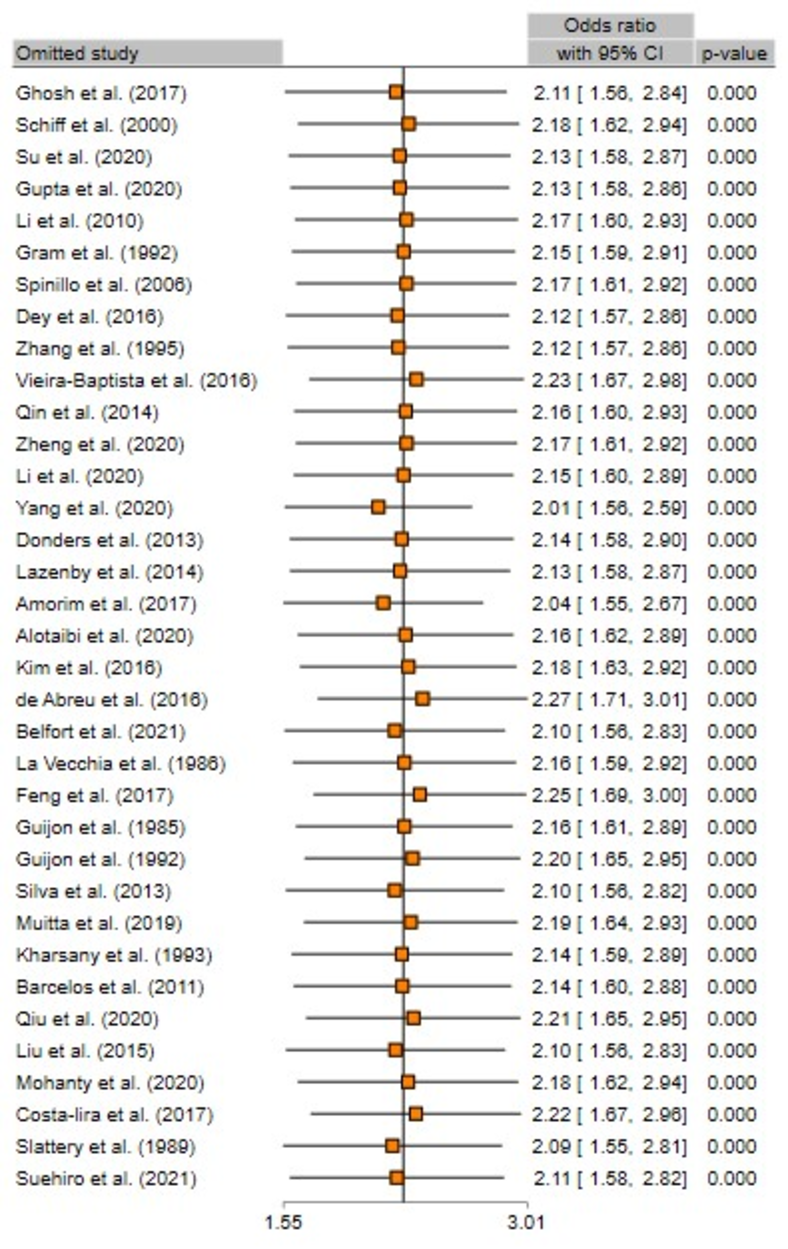

Supplement: S2 Fig — (TIF) [file pone.0288443.s002.tif]

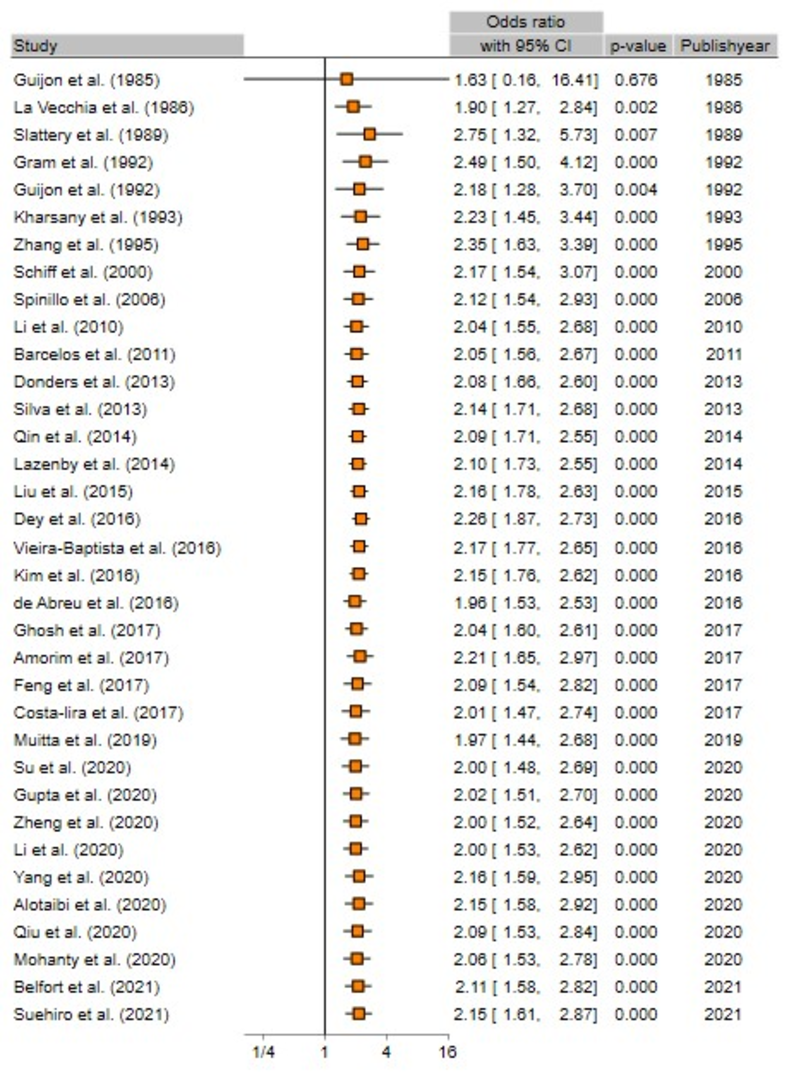

Supplement: S3 Fig — (TIF) [file pone.0288443.s003.tif]

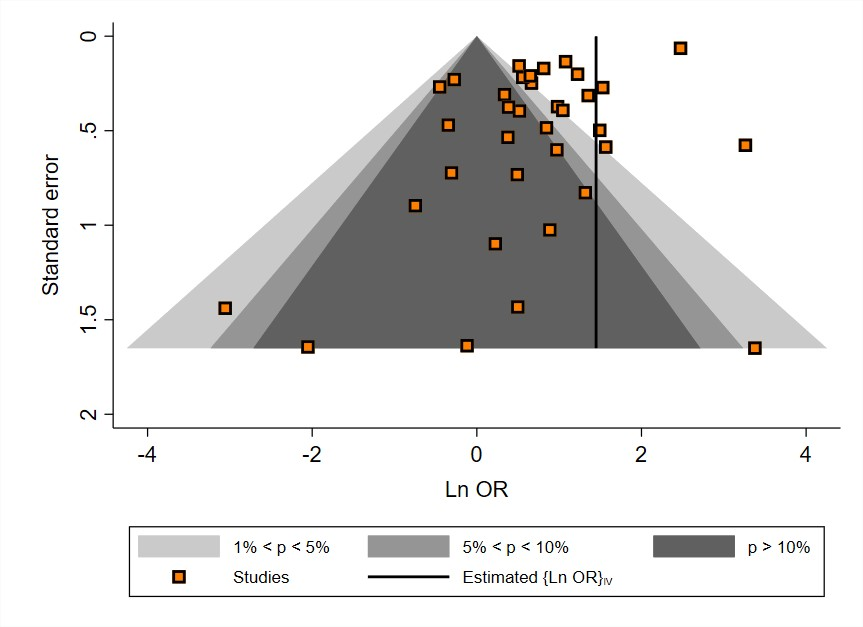

Supplement: S4 Fig — (TIF) [file pone.0288443.s004.tif]

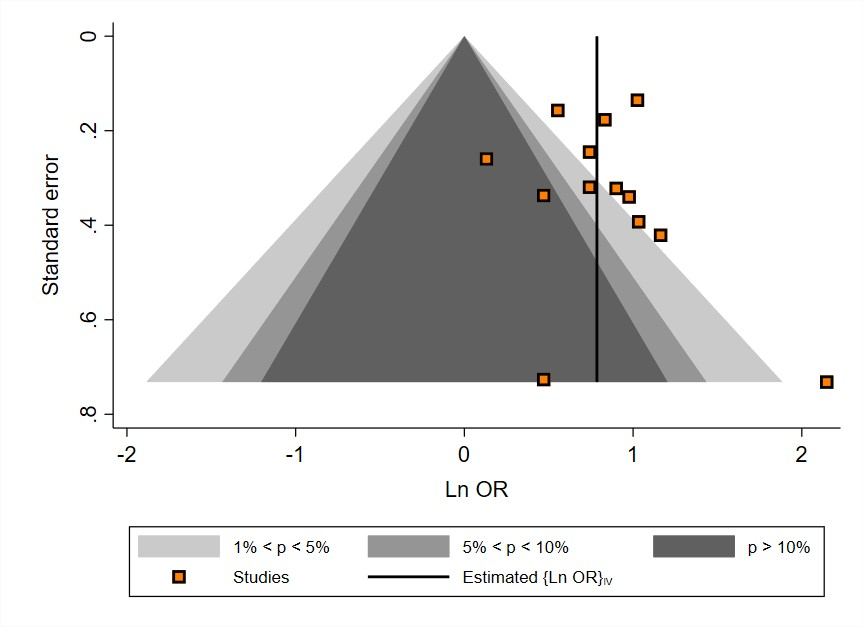

Supplement: S5 Fig — (TIF) [file pone.0288443.s005.tif]

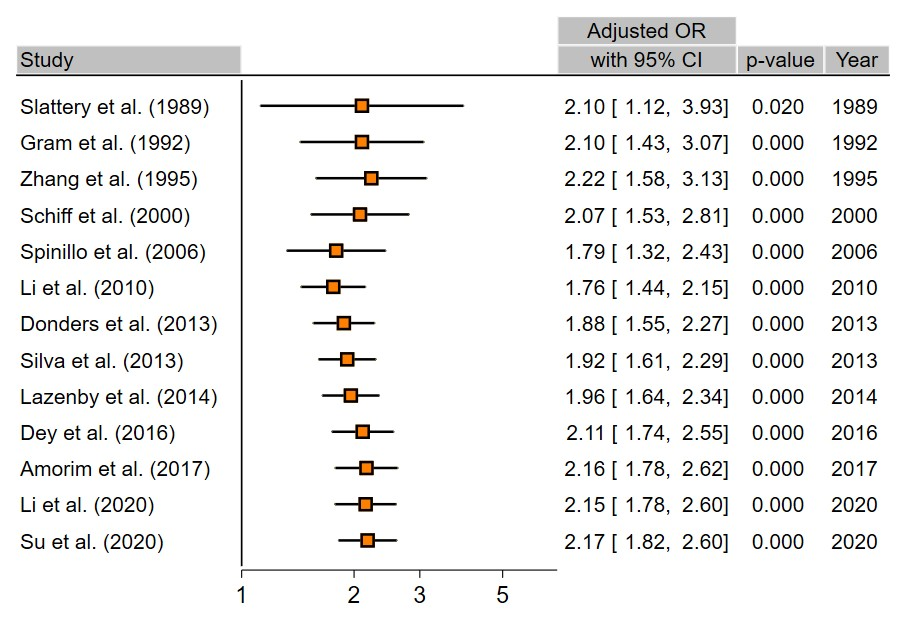

Supplement: S6 Fig — (TIF) [file pone.0288443.s006.tif]

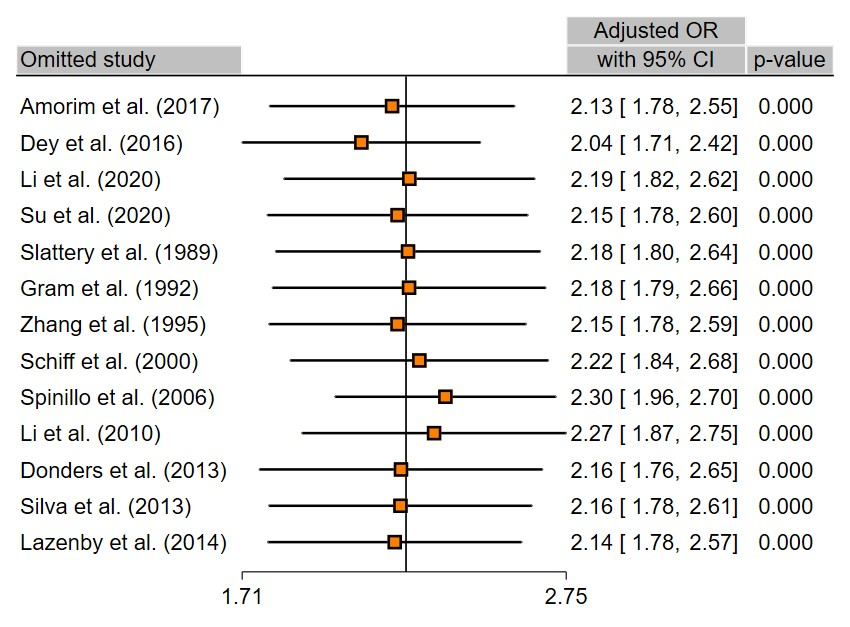

Supplement: S7 Fig — (TIF) [file pone.0288443.s007.tif]
